# Oligoprogression in Non-Small Cell Lung Cancer

**DOI:** 10.3390/cancers13225823

**Published:** 2021-11-20

**Authors:** Daijiro Harada, Nagio Takigawa

**Affiliations:** 1 Department of Thoracic Oncology, National Hospital Organization Shikoku Cancer Center, Matsuyama 791-0280, Japan; 2 Department of General Internal Medicine 4, Kawasaki Medical School, Okayama 700-8505, Japan

**Keywords:** non-small cell lung cancer, oligometastatic disease, oligoprogressive disease, driver mutations, tyrosine kinase inhibitor, immune checkpoint inhibitor, local ablative therapy, stereotactic body radiotherapy

## Abstract

**Simple Summary:**

Several retrospective studies present evidence of oligoprogressive disease (OPD) in patients with non-small cell lung cancer (NSCLC) with driver mutations such as EGFR. The strategy of local ablative therapy (LAT) with radiotherapy, followed by the continuation of the same anticancer drug therapy beyond progression disease, is recommended in the current NCCN guideline. Although evidence of the use of this strategy in the treatment of the driver mutation-negative NSCLC is missing, LAT with radiotherapy for OPD after combination therapy of immune checkpoint inhibitor with cytotoxic chemotherapy is expected. Tumors outside of the radiation field may further respond to the immune checkpoint inhibitors due to an abscopal effect. In the future, to achieve long-term survival in advanced NSCLC, it will be important to validate this treatment strategy via prospective comparative studies and to actively implement it in clinical practice.

**Abstract:**

We reviewed the literature on oligoprogressive disease (OPD) and local ablative therapy (LAT) in patients with advanced non-small cell lung cancer (NSCLC). The frequency of OPD varies depending on its definition and is estimated to be between 15–47%. The implications of the strategy of continuing the same anticancer agents beyond progressive disease after LAT with radiation therapy for OPD are based on the concept of progression in which only a small number of lesions, not more than about four, proliferate after chemotherapy. In the case of OPD harboring driver mutations such as EGFR, prospective studies are underway. However, evidence from retrospective studies support this strategy, which is currently recommended in some guidelines. The prognosis in OPD cases during the administration of an immune checkpoint inhibitor (ICI) is relatively promising. Additionally, LAT with radiation for OPD after the first-line treatment of ICI with cytotoxic chemotherapy may overcome the resistance to the combination drug therapy due to an abscopal effect. To achieve long-term survival in advanced-stage NSCLC, it is important to verify the optimal method and timing of the therapy through prospective comparative studies as well as patient selection based on patient characteristics and biomarker levels.

## 1. Introduction

The concept of oligometastatic disease (OMD) was first defined in an editorial by Hellman et al. in 1995, and it placed cancer in an intermediate state between the localized early stage and the advanced metastatic stage [1]. In principle, patients with OMD are classified as M1 in the TNM classification due to the presence of metastasis, which corresponds to stage IV. Thus, the standard treatment to prolong the overall survival time is chemotherapy in the case of metastatic non-small cell lung cancer (NSCLC).

However, with the improvement of imaging techniques, the development of effective chemotherapy, and the improvement of surgical and radiotherapeutic techniques, patients treated with local ablative therapy (LAT) for OMD lesions seem to have a better prognosis than those treated with chemotherapy alone for multiple metastatic lesions. Therefore, it is necessary to establish a new classification of the advanced stage of cancer and standard treatment strategies. Most reports on the results of LAT for OMD are based on retrospective studies. Moreover, even among the few prospective studies reporting on LAT for OMD, the definition of OMD varies and includes 3–5 lesions [2]. First, it is important to understand the definitions of OMD in such studies and to interpret the effectiveness of LAT.

Oligoprogressive disease (OPD), which is included in the OMD classification, has not been firmly established but is a concept of progression in which only a small number of lesions develop after induction chemotherapy and the primary or other metastatic sites are well-controlled [3,4]. In these cases, heterogeneity in the systemic tumor lesions may render the OPD lesions resistant to induction chemotherapy [5].

The Delphi process was conducted by 20 international experts, including 19 members of the European Society for Radiotherapy and Oncology and the European Organization for Research and Treatment of Cancer OligoCare project. This classification is useful for the future analysis of the evidence for OMD, for the consideration of treatment strategies in clinical practice, and for the conduct of clinical trials. This classification is based on the biological and clinical history of OMD and applies to all types of carcinomas [2].

In this review, we searched scientific databases, PubMed and clinicaltrials.gov, for recent topics related to OPD and LAT in NSCLC and conducted a review based on the classification of the consensus. This classification of OMD is based on the following four perspectives: (1) history of polymetastatic disease before a diagnosis of oligometastatic disease; (2) prior history of oligometastatic disease; (3) the first diagnosis of oligometastatic disease; and (4) synchronous and metachronous oligometastatic disease. Based on the answers to the five questions, the OMDs found in the images were classified into nine categories: (1) synchronous oligometastatic disease; (2) metachronous oligorecurrence; (3) metachronous oligoprogression; (4) repeat oligorecurrence; (5) repeat oligoprogression; (6) repeat oligopersistence; (7) induced oligorecurrence; (8) induced oligoprogression; and (9) induced oligopersistence. In this paper, we reviewed the literature on OPD cases that fit the two definitions: “receiving aggressive systemic therapy at the time of diagnosis of oligometastatic disease” and “any oligometastatic disease that has progressed on current imaging” and the implications of the strategy of continuing the same treatment beyond the progression of the disease after LAT.

## 2. Sites of OPD

The literature on LAT for OPD is mainly based on the reports of cases with EGFR gene mutation and ALK fusion gene mutation. Table 1 summarizes the data available in the literature, and the sites of OPD are listed. Although the frequency of OPD sites varied from report to report, OPD tended to be more common in the central nervous system (CNS), intrapulmonary lesions, lymph nodes (LNs), and bones, and less common in adrenal and liver metastases.

## 3. OPD in NSCLC with Driver Mutations

The progression pattern can be classified into three categories: (1) progression of CNS metastasis alone; (2) systemic progression with or without CNS; and (3) OPD [4]. The frequency of OPD varies depending on its definition and is estimated to be between 15–47% [4,7,15].

For the treatment of OPD in patients with NSCLC with driver mutation, the addition of LAT, such as stereotactic body radiotherapy (SBRT), while continuing the previous tyrosine kinase inhibitor (TKI) may be adopted. This is because SBRT is preferred over conventional radiotherapy (RT) or invasive surgical treatment as it requires less time for interruption of TKI. As for other LATs, surgery is an important treatment modality for resection of primary and metastatic lesions in OMD, and retrospective studies have revealed beneficial results of LATs in patients with OPD [16]. However, surgical invasion and acute complications may increase the risk of prolonged resumption time for subsequent TKI therapy beyond PD. A retrospective analysis reported that radiofrequency ablation (RFA) was applied to lung lesions in 2 of 46 OPD cases [8]. Furthermore, a retrospective analysis of cryotherapy for OPD showed survival benefits [17]. However, RFA and cryotherapy tend to have high incidences of complications, such as hemoptysis and pneumothorax/pleural effusion requiring drainage [18,19]. Thus, to standardize surgery, RFA and cryotherapy as LAT for OPD, issues such as the relatively high possibility of prolonging resumption time of subsequent therapy beyond PD and the bias in treatment outcomes among different physicians and facilities need to be considered and resolved [20].

In retrospective analysis, the addition of LAT to OPD, mainly during treatment with molecularly targeted agents, is expected to improve local control, suppress secondary metastasis, and prolong progression-free survival (PFS) and overall survival (OS) (Table 2) [6,7,8,9,10,11,12,13,21,22,23]. The median PFS in patients with NSCLC with driver mutation treated with LAT is generally expected to be 6 months or more, and there are reports of significantly longer overall survival than in patients who are not eligible for LAT.

Multivariate analysis in a retrospective study by Qiu et al. showed that more than 6 months between the onset of OPD and the initiation of LAT was associated with better long-term PFS and OS. This may suggest that a certain period of observation is important to determine whether a patient is ready to be transferred to systemic PD and to select patients with OPD who will benefit from LAT [8].

In a multivariate analysis of the retrospective study by Xu et al., four factors including female sex, *EGFR* exon 19 deletion mutation, single OPD, and a good response to EGFR-TKI were independent predictors of PFS1 (defined as the time from initiation of TKI therapy to progressive disease (PD) or death) and PFS2 (defined as the time from initiation of TKI therapy to off-TKI PD), and OS [13].

Multivariate analysis in a retrospective study by Friedes et al. showed that good performance status (PS), low number of metastases at diagnosis (≤3), time from the start of TKI to the onset of OPD longer than 6 months, and first- or second-line treatment were positive predictors of radiological PFS. The more foci of OPD (more than two), the worse the radiological PFS, defined from the beginning of RT to any documented radiological progression at any site [22]. Based on the results of these analyses, the strategy of continuing the same treatment beyond the PD after LAT might be suggested for patients with good PS, a certain duration of response (6 months or longer) to TKI, and without progression to systemic PD after the occurrence of OPD. In other words, OPD patients with these characteristics would benefit clinically from the continuation of the current TKI.

Since these studies are retrospective, there could be bias due to variations in patient backgrounds [10,23]. However, the hazard ratios (HR) are clinically non-negligible, which may provide a rationale for the active consideration of LAT in OPD in patients with NSCLC with driver oncogene mutations.

Although most retrospective studies suggested that a strategy of controlling TKI-resistant OPD by aggressive LAT is clinically beneficial, the following theoretical basis can be considered. LAT will aid in the prevention or treatment of symptoms and complications caused by growing tumors in the near future, may prevent secondary dissemination of TKI-resistant clones, and may allow the continuation of current TKI maintenance therapy [24]. For example, SBRT to asymptomatic brain metastasis in OPD may delay cranial symptoms and allow continuation of TKI therapy until systemic metastases, resulting in a survival benefit.

Based on the retrospective studies mentioned above, the U.S. National Comprehensive Cancer Network guidelines currently recommend a strategy of employing LAT for OPD [25]. A prospective study in this regard is also underway, and the results will clarify the evidence in the future [12]. HALT (NCT03256981) is a multicenter phase II/III study of 110 patients with advanced NSCLC with driver mutations who developed OPD after induction of TKI therapy [11]. The study design was to randomize patients to receive SBRT with continued TKI therapy or standard chemotherapy for up to three extracranial sites of OPD and to compare PFS as the primary endpoint.

## 4. OPD in NSCLC without Driver Mutations

There are few reports on the efficacy of continuing treatment beyond the PD after the addition of LAT for OPD in NSCLC without driver mutations. The ongoing STOP-NSCLC (NCT02756793) is a randomized, multicenter, phase II trial with a primary endpoint of PFS in patients with OPD (up to three lesions in a single organ, including the brain, for a total of up to five lesions) in NSCLC without driver mutations, comparing SBRT for OPD and continuation of current systemic therapy versus standard chemotherapy [26].

NCT03808662 is an open-label, randomized phase II study in patients with triple-negative breast cancer and stage IV NSCLC without *EGFR/ALK* mutation. The primary endpoint was the PFS, and the secondary endpoint was the OS. Characteristically, this trial is open to patients with medical conditions that preclude participation in other systemic therapies or drug trials. The purpose of this study was to determine whether receiving SBRT, when the subject’s metastatic tumor (up to five lesions, excluding the brain) is just beginning to grow, will prolong the time until the worsening of the disease. STOP-NSCLC, HALT, and NCT03808662 trials examined the efficacy and safety of SBRT of all progressions in patients with OPD after response to systemic chemotherapy, and the results of these trials may allow us to evaluate the efficacy and safety of a strategy of continuation of treatment beyond PD with the addition of LAT in OPD, with and without driver mutations.

## 5. OPD in NSCLC Patients Treated with Immune Checkpoint Inhibitor

Patients with *EGFR* and *ALK* mutations have been excluded from the clinical trials of combination therapy of immune checkpoint inhibitor (ICI) with cytotoxic chemotherapy due to their low efficacy, and the development of treatment has been mainly focused on patients with NSCLC without driver mutations. ICI with and without cytotoxic chemotherapy has also been reported to have a favorable prognosis in patients with OPD.

Rheinheimer et al. defined OPD as a localized treatment failure at one or two anatomic sites, with one to five progressive measurable lesions (according to RECIST 1.1). As for the characteristics of the development of OPD, when ICI monotherapy was compared between first- and second-line therapy, it was shown to be more frequent in first-line therapy (20 vs. 10%, *p* < 0.05), to occur at a later time (median 11 vs. 5 months, *p* < 0.01), to affect fewer organs (mean 1.1 vs. 1.5, *p* < 0.05), and to have fewer lesions (1.4 vs. 2.3, *p* < 0.05). The frequency by organ was 42% for mediastinal LNs, 39% for the brain, and 24% for the lung. Compared with multiple PD, OPD had a later onset (9 vs. 2 months, *p* < 0.001), a better prognosis (mean 26 vs. 13 months, *p* < 0.001), and more cases with high PD-L1 expression (*p* < 0.001) [27].

Heo et al. analyzed 125 patients treated with ICI and reported that 63 (50.4%) developed acquired resistance (AR) (exacerbation after more than 6 months of treatment) at a median of 10.7 months. Patients were treated with PD-1/PD-L1 inhibitor monotherapy (82.5%), PD-1/PD-L1 inhibitor plus CTLA−4 inhibitor (15.8%), and ICI plus chemotherapy (1.6%). Patients with *EGFR* (17.4%) and *ALK* (1.6%) mutations were also included. Of the 63 AR patients, 52 (82.5%) had OPD (defined as up to 2 lesions), and OPD patients had a better OS than those with the multiple progressive diseases (MPD) (3 or more lesions) (18.9 vs. 8.8 months, *p* = 0.04). In the analysis in OPD, OS was significantly better in patients with OPD in extra-thoracic lesions other than the liver: 30.2 months, intra-thoracic OPD: 11.7 months, and OPD in the liver: 5.4 months (*p* < 0.001). However, it was also suggested that the content of subsequent treatment (ICI or chemotherapy) may not be related to OS (*p* = 0.723) [28].

Kagawa et al. reported a retrospective observational study of 148 cases of NSCLC, including driver mutation-positive cases. Thirty-eight patients developed OPD, and univariate analysis showed that the frequency of OPD was higher in patients who responded to ICI and had PFS longer than 6 months. OPD occurred most frequently in the primary site in 15 cases, followed by abdominal LN in six cases. Of the abdominal LN metastases, four were new lesions. PFS was significantly longer in patients with OPD than in those with MPD (median PFS: 7.37 vs. 2.50 months, *p* < 0.0001), and OS was also significantly longer in patients with OPD than in those with MPD (not reached vs. 12.94 months, *p* < 0.0001). However, the LAT did not affect the OS for OPD [14].

Hosoya et al. performed a retrospective analysis of 174 patients with a tumor proportion score ≥50% and who received initial pembrolizumab therapy. Progression after response was defined as AR, and five or fewer progression lesions were defined as OPD. Eighty-eight patients responded, 46 of whom had AR. In univariate analysis, PFS was shorter in the elderly (≥75 years), those with poor PS (2–4), those in whom the number of metastatic organs was ≥3, and those with bone metastasis. Of the 46 patients, 32 (70%) had OPD and had significantly longer post-progression survival than non-OPD patients: 16.2 months (95% confidence interval [CI]: 11.5—not reached) vs. 11.5 months (95% CI: 2.5–not reached) (HR: 0.31, 95% CI: 0.11–0.92, *p* = 0.035). Of the OPD patients, 7 (15%) underwent LAT with RT for all lesions, and of these, the median 2nd PFS in the 4 patients who continued treatment with pembrolizumab did not reach beyond the PD (95% CI: 7.7 months—not reached) [29].

These retrospective analyses suggest that the incidence of OPD in patients with ICI is approximately 20%, the median time of onset is 7–9 months, and the prognosis in OPD is better than that in MPD. OPD sites were reported relatively frequently in the primary tumor, mediastinal LN, brain, and intrapulmonary and abdominal LNs and tended to be more frequent in patients with response to ICI, patients with PFS longer than 6 months, males, patients with *EGFR* wild-type mutation, and patients with a smoking history. However, the impact of the LAT on the OS is uncertain. In addition, these data are from retrospective observational studies and should be interpreted with caution, keeping in mind that there are various biases and differences in the definition of OPD (number of OPD, response to ICI, and minimum duration of treatment). Since the efficacy of ICI is higher in the first-line than in the later-line regimens, evidence from standard first-line treatment including ICI should be emphasized for OPD [30,31].

Yamaguchi et al. showed in a retrospective observational study that pretreatment with radiation significantly improved the response rate and PFS of ICI treatment [32]. In a randomized controlled phase II trial, Theelen et al. reported that application of pembrolizumab after SBRT to a single tumor had a trend towards a better overall response rate (ORR), PFS, and OS at 12 weeks compared with pembrolizumab alone [33], suggesting that LAT with RT may be a clinically useful strategy to increase the chance of long-term survival in patients with OPD after initial treatment with ICI plus chemotherapy.

As for the mechanism by which radiation for OPD may enhance the clinical efficacy of ICI, as well as the evidence for the efficacy of LAT in patients with driver mutation, it has been shown that besides prolonging PFS2 by controlling OPD, radiation may also contribute to this strategy by modifying cell surface molecules to enhance the efficacy of immunotherapy, activating the innate immune system to produce an abscopal effect, and enhancing the efficacy of ICI by reducing the tumor volume [34,35,36].

Currently, there are several prospective clinical trials of LAT for OPD after ICI therapy in NSCLC and for continuation of ICI therapy beyond the PD. For example, the OLCSG 2001 [UMIN000041778] study is a single-arm phase II study that evaluates the efficacy and safety of adding localized radiation as LAT and continuing maintenance therapy beyond the PD in patients who develop OPD after initial immune-chemotherapy. The primary endpoint is 1-year survival rate, and the secondary endpoints are OS, PFS, safety, and post-treatment status. NCT04767009 is an open-label, multicenter, phase II study to evaluate the efficacy and safety of SBRT in patients with OPD, who were defined as those not requiring palliative irradiation and whose numbers of OPD are based on the opinion of the investigator, in NSCLC without driver mutation after an anti PD-1 inhibitor, followed by PD-1 inhibitor maintenance beyond the PD. The primary endpoints are safety and 1-year new lesion-free survival rate, and the secondary endpoints are PFS and OS. NCT04549428 is a multicenter, open-label, single-arm phase II study to evaluate the preliminary efficacy, safety, and tolerability of atezolizumab in combination with 8-Gy single-dose radiation therapy in patients who were diagnosed with stage IV NSCLC with OPD. The study considered patients who had up to four OPD lesions in up to three organs, excluding the brain and bone, with both anti-PD-1 agents and first-line cytotoxic chemotherapy, regardless of PD-L1 status. The primary endpoint is the ORR. NCT04517526 is a multicenter, phase II study to evaluate the efficacy and safety of platinum-based chemotherapy + bevacizumab + durvalumab and salvage SBRT for patients with stage IV NSCLC with *EGFR* mutations after the failure of first-line osimertinib. The primary endpoints are the PFS and OS.

When positive results are obtained in the above studies, randomized phase 3 studies to confirm the long-term effectiveness of LAT should be performed on a large scale.

## 6. Limitations

This review has some limitations. One of the points to be noted when using evidence of OPD is the difficulty in making a unified interpretation because each report has a different format for reporting survival, a different control group, and a different definition of OPD. In the retrospective studies, the control groups are usually the groups in which LAT was not indicated. Many patients were clinically judged to be ineligible for LAT, and the existence of selection bias makes it difficult to appropriately evaluate OS. In addition, since patients who were able to undergo LAT seem to have a relatively good prognosis, comparing the efficacy of continuing the current treatment beyond the PD after LAT with that of the historical control does not allow us to determine the true efficacy.

Second, RT has not been established as a means of intervention for OPD. Therefore, the method and timing of RT are often determined by the attending physicians and radiation oncologists. As for the toxicity of RT, the incidence of radiation necrosis as a central nervous system toxicity has been reported to be 5–25%, including asymptomatic cases [37,38]. Among patients with OMD, severe pulmonary and esophageal toxicities were reported in 6.4% and 19.4% of patients, respectively [39]. Among patients who underwent thoracic RT, possibility of enhancement of cardiopulmonary toxicity with increased RT dose to the heart and shortening of overall survival were reported [40]. Among patients undergoing ICI, particular attention should be paid to pulmonary toxicity as the possibility of increased frequency of pneumonitis in sequential ICI treatment has been reported [41]. Additionally, there is concern about radiation recall pneumonitis due to chemotherapy in the late stage [42]. However, in general, RT for OPD had a low incidence of serious complications in the previously described trials and hence, can be considered a relatively safe and well-tolerated LAT. The irradiated field for OPD might be narrower than that for OMD because LAT is performed only on enlarged lesions after assessing its response to previous treatment. Thus, RT for OPD can be considered less toxic. There is little clear evidence that has been validated on the optimal dosage and timing of RT, considering the balance between anti-tumor effect and toxicity, and it is necessary to validate the optimized method of RT in clinical trials.

Third, evidence from prospective phase II trials often showed only PFS results. The effect of prolonging OS needs to be evaluated.

Fourth, enrollment in randomized controlled trials to validate the treatment of OPD with LAT, followed by a continuation of current therapy beyond the PD, is difficult because many eligible patients are needed. The maximum number of patients recruited in the above-mentioned and ongoing phase II studies was 60 for NCT04517526. Large multicenter research is necessary to accomplish a randomized study.

## 7. Summary and Conclusion

Concerning the efficacy and safety of LAT against OPD, prospective comparative studies have provided some evidence. Several studies, including retrospective studies, defined the number of OPD lesions as either ≤3 [7,9,11,14,22] or ≤5 [8,12,13,23]. Two studies excluded cases of metastasis to the central nervous system [6,21] and one included cases in which LAT was performed and did not specify the number of lesions [22]. Thus, most studies define the number of OPDs as ≤5 and the number of organs as ≤3. Therefore, standardization in the definition of OPD is an important issue. To identify patients who would benefit from LAT and could continue current therapy beyond PD, it is also important to select patients who would not rapidly progress to multiple metastases. From the reports described above, in patients with driver mutations, an appropriate OPD case may be defined as one who can continue the current treatment beyond PD for about six months from the time of progression to the start of LAT. Concerning cytotoxic chemotherapy, patients with PFS for more than six months might be considered appropriate for the requirement of OPD to benefit from LAT and subsequent continuation of the current chemotherapeutic drugs beyond PD. However, it is unclear whether these definitions are true for ICI treatment.

Currently, ICI treatment has become the standard of care, and radiation therapy is expected to improve the efficacy of ICI by producing an abscopal effect and reducing tumor volume. This mechanism is expected to have a beneficial effect on LAT and subsequent continuation of the ICI beyond PD for OPD. Long-term survival in patients treated with ICI plus chemotherapy has been observed at a certain percentage. Hopefully, a multidisciplinary treatment strategy using ICI, cytotoxic agents, molecular-targeted agents, and radiation will reveal the possibility of long-term survival or cure in patients with metastatic NSCLC who do not respond adequately to ICI plus chemotherapy. There is also a need to establish clinically useful biomarkers to screen patients with rapid disease progression. Biomarkers in serum/plasma and tumor micro-environment may help detect the disease state [34].

In daily clinical practice, some patients meet the definition of OPD in clinical trials. They do not always receive LAT or subsequent continuation of the current therapy beyond PD. This is because established definitions and evidence of OPD are missing. It is important to conduct prospective comparative studies to verify the efficacy and safety, establish appropriate eligibility criteria, identify biomarkers, and determine the optimal dose and timing of LAT.

## Figures and Tables

**Table 1:** Sites of oligoprogressive disease.

Authors	Driver Mutation	Design	*n*	CNS	(%)	Lung	(%)	Lymph Node	(%)	Bone	(%)	Adrenal Gland	(%)	Liver	(%)	Other		(%)	Ref
Yu et al.	EGFR m+	retro	18	3	(17)	14	(78)	8	(44)	4	(22)			1	(6)	1	spleen	(6)	[6]
Weickhardt et al.	ALK rearrange. EGFR m+	retro	25	13	(52)	7	(28)	2	(8)	7	(28)	2	(8)	1	(4)				[7]
Qiu et al.	EGFR m+	retro	46	24	(52)	16	(35)			6	(13)								[8]
Chan et al.	EGFR m+	retro	46	5	(11)	18	(39)	2	(4)	3	(7)	1	(2)			1	pancreas	(2)	[9]
Rossi et al.	EGFR m+	retro	30	8	(27)	9	(30)	8	(27)	11	(36)			1	(3)				[10]
Santarpia et al.	EGFR m+	retro	36	9	(25)	11	(31)	9	(25)	5	(14)	2	(6)						[11]
Weiss et al.	EGFR m+ at least 6 months without PD	P2	25	2	(8)	15	(60)			7	(28)			4	(16)				[12]
Xu et al.	EGFR m+	retro	206	124	(60)	40	(19)	9	(4)	86	(42)	35	(17)	18	(9)	11	chest wall, intestine	(5)	[13]
Kagawa et al.	NSCLC m+ or WT	retro	10	1	(10)			6	(60)	1	(10)	1	(10)			1	gall bladder	(10)	[14]

CNS: central nervous system; NSCLC: non-small cell lung cancer; m+: mutation; P2: phase II study; PD: progression disease; retro: retrospective study; WT: wild type.

**Table 2:** Outcomes of local ablative therapy for oligoprogressive disease.

Authors	Driver Mutation	Design	*n*	Induction Tx	Intervention	Mainte. Tx	mPFS1 (m)	TTP from Intervention (m)	Duration of Treatment (m)	MST (m)	MST from Intervention (m)	Comment	Ref
Gan et al.	ALK fusion+	retro	14	crizo	local ablative Tx (RT or OP)	crizotinib	14	5.5	28	NA	NA		[21]
			13		not eligible for LAT		7.2	NA	NA				
Yu et al.	EGFR m+	retro	18	gef or erlo	local ablative Tx (RT or OP)	gefitinib or erlotinib	19	10.0	NA	NA	41.0	TTF from intervention: 22 m	[6]
Weickhardt et al.	ALK fusion+ (*n* = 38) EGFR m+ (*n* = 27)	retro	ALK rearrange (*n* = 15) EGFR m+ (*n* = 10)	crizo erlo	local Tx (RT or OP)	crizotinib erlotinib	9.0 12.0	6.2	NA	NA	NA		[7]
			26	crizo or erlo	Pts without LAT		12.8	NA					
Qiu et al.	EGFR m+	retro	46	EGFR-TKI	local ablative Tx (RT or RFA)	EGFR-TKI	NA	7.0	NA	35.0	13.0		[8]
Chan et al.	EGFR m+	retro	25	EGFR-TKI	local ablative Tx (RT)	same TKI	NA	7 *^1^	NA	NA	28.2 *^2^	*^1^: *p* = 0.0017. *^2^: HR:0.4 4[95%CI:0.21–0.92], *p* = 0.030	[9]
			25 (matched cohort)		chemotherapy			4.1			14.7		
Rossi et al.	EGFR m+	retro	30	EGFR-TKI	local ablative Tx (RT)	same TKI	13.8	6.7 *^3^	NA	37.3 *^4^	NA	*^3^: HR: 0.54 [95%CI:0.24–1.18], *p* = 0.06. *^4^: *p* < 0.0001. *^5^: *p* = 0.0015	[10]
			13		same TKI		12.3	3.1		20.1			
			88		2nd line treatment or BSC		8.9	NA		15.1			
	EGFR m+ without intrinsic resistance to EGFR-TKI	retro	29	EGFR-TKI	local ablative Tx (RT)	same TKI	NA			37.3 *^5^	NA		
			12		same TKI			NA	NA	20.1			
			64		2nd line treatment or BSC					21.9			
Santarpia et al.	EGFR m+	retro	36	EGFR-TKI	high-dose radiation therapy	EGFR-TKI	12.5	6.3		38.7			[11]
Schmid et al.	EGFR T790M+	retro	13	osim	local ablative Tx (RT or OP)	osimertinib	NA	6.7	19.6	28.0	NR *^6^	*^6^: *p* = 0.2	[23]
			13		osimertinib						20.2		
Weiss et al.	EGFR m+ at least 6 m without PD	P2	25	erlo	SRT	erlotinib	NA	6.0	NA	NA	29.0		[12]
Xu et al.	EGFR m+	retro	206	EGFR-TKI	local ablative Tx (RT or OP)	EGFR-TKI	10.7		18.3	37.4			[13]
Friedes et al.	NSCLC	retro	253	chemo or TKI	definitive RT	same systemic Tx	NA	7.9	NA	NA	NA	TTF from intervention was 8.8 months.	[22]
Kagawa et al.	NSCLC	retro	10	ICI	local ablative Tx (RT or OP)	ICI beyond PD (*n* = 6)	10.4	NA	NA	NA	NR *^7^	*^7^: *p* = 0.456	[14]
			28		no local therapy		NA				NR		

BSC: best supportive care; chemo: chemotherapy; CNS: central nervous system; crizo: crizotinib; erlo: erlotinib; gef: gefitinib; ICI: immune checkpoint inhibitor; NA: not available; NR: not reached; NSCLC: non-small cell lung cancer; m+: mutation; m: month; mainte.: maintenance; MST: median survival time; OP: operation; osim: osimertinib; P2: phase II study; PD: progression disease; retro: retrospective study; RT: radiation therapy; TKI: tyrosine kinase inhibitor; TTF: time to treatment failure; TTP; time to progression; Tx: treatment; WT: wild type. The * superscript numbers indicate the test of comparison of each outcome in the corresponding clinical trials.

## Data Availability

The dataset on which this study was based is available from the corresponding author upon reasonable request.
